# Supplementary material for: A Likelihood Approach for Real-Time Calibration of Stochastic Compartmental Epidemic Models
Source: PLoS Comput Biol. 2017 Jan 17;13(1):e1005257. doi: 10.1371/journal.pcbi.1005257 (PMC5240920; doi:10.1371/journal.pcbi.1005257)
Supplement: S1 File — (TAR.GZ) [file pcbi.1005257.s014.tar.gz › HSPH_Online-SI-Revision/Additions/EnKF08/n100-extreme-SEITR_noisy/n100-extreme-SEITR_noisy_EnKF2_table-8.pdf]

1	newly 3 weeks spec	2107.88	1817.72	{47., 5198.}	108.544	0.712913	newly 3 weeks cumul	3792.89	10 135.8	{2653., 35 607.}	189.278	0.28117	newly 5 weeks spec	7087.2	535.52	{1., 1299.}	93.3082	1.88614	newly 5 weeks cumul	15 036.4	11 668.2	{3139., 39 478.}	91.6763	0.533139	newly 58 weeks cumul	94 514.	12 441.4	{3383., 40 843.}	87.155	1.14239
2	newly 3 weeks spec	265.	9104.31	{403., 21 007.}	3341.77	1.38017	newly 3 weeks cumul	343.	50 263.6	{16 467., 90 309.}	14 554.1	2.07864	newly 5 weeks spec	3034.	4426.98	{0., 8945.}	99.1411	0.854616	newly 5 weeks cumul	4334.	61 021.5	{24 394., 113 519.}	1307.97	1.08685	newly 66 weeks cumul	99 017.	72 531.2	{33 227., 114 901.}	35.8997	0.216714
3	newly 3 weeks spec	257.	6576.46	{335., 18 170.}	2471.4	1.21801	newly 3 weeks cumul	379.	35 026.7	{5974., 86 203.}	9142.3	1.75277	newly 5 weeks spec	2684.	3920.72	{1., 12 286.}	126.137	1.06696	newly 5 weeks cumul	3894.	44 003.7	{7770., 90 759.}	1033.85	0.907293	newly 68 weeks cumul	98 993.	57 268.9	{9064., 99 207.}	43.7854	0.401988
4	newly 3 weeks spec	698.	11 504.	{2253., 23 142.}	1559.62	1.13824	newly 3 weeks cumul	1030.	53 402.6	{18 975., 88 305.}	5084.71	1.62684	newly 5 weeks spec	3933.	4899.47	{230., 9777.}	78.3455	0.617675	newly 5 weeks cumul	6733.	65 846.9	{21 544., 105 192.}	878.34	0.917854	newly 66 weeks cumul	96 862.	76 366.9	{30 376., 111 630.}	34.3168	0.216497
5	newly 3 weeks spec	187.	6615.11	{235., 16 130.}	3452.65	1.3938	newly 3 weeks cumul	237.	38 122.9	{4470., 89 442.}	15 985.6	1.98481	newly 5 weeks spec	3231.	4405.04	{1., 12 452.}	136.601	1.37922	newly 5 weeks cumul	4188.	47 928.4	{5791., 89 499.}	1052.74	0.931297	newly 68 weeks cumul	98 880.	62 229.1	{6414., 99 287.}	37.3797	0.374721
6	newly 3 weeks spec	323.	11 610.	{1453., 20 555.}	3497.39	1.44104	newly 3 weeks cumul	460.	46 663.1	{13 624., 95 161.}	10 044.1	1.90906	newly 5 weeks spec	3690.	6117.09	{147., 12 215.}	112.75	0.632047	newly 5 weeks cumul	5281.	61 390.	{17 333., 96 787.}	1062.47	0.989462	newly 67 weeks cumul	95 041.	76 563.	{22 695., 108 622.}	26.5999	0.186159
7	newly 3 weeks spec	2336.	1305.4	{125., 598.}	114.772	0.920851	newly 3 weeks cumul	4235.	5742.41	{1418., 4947.}	134.602	0.412974	newly 5 weeks spec	6848.	460.52	{11., 285.}	94.9761	1.88766	newly 5 weeks cumul	15 415.	6967.68	{1678., 5020.}	101.339	0.828176	newly 59 weeks cumul	97 367.	7899.26	{1682., 5020.}	91.9893	1.50006
8	newly 3 weeks spec	476.	10 590.5	{2404., 21 505.}	2132.3	1.22317	newly 3 weeks cumul	697.	49 667.2	{15 295., 89 384.}	7025.85	1.7043	newly 5 weeks spec	4729.	4554.9	{109., 9789.}	62.3578	0.501321	newly 5 weeks cumul	7052.	61 136.5	{19 521., 110 424.}	776.202	0.888122	newly 66 weeks cumul	99 432.	71 787.3	{23 709., 123 887.}	39.475	0.301949
9	newly 3 weeks spec	88.	7717.33	{561., 22 143.}	8674.22	1.6629	newly 3 weeks cumul	112.	45 836.9	{7459., 95 151.}	40 825.8	2.42254	newly 5 weeks spec	2227.	3634.67	{3., 10 092.}	148.065	1.14103	newly 5 weeks cumul	2748.	54 817.2	{9579., 96 043.}	1894.8	1.15318	newly 68 weeks cumul	99 354.	66 647.4	{14 715., 100 555.}	33.9162	0.269581
10	newly 3 weeks spec	982.	7370.36	{912., 20 603.}	653.904	0.680135	newly 3 weeks cumul	1566.	48 455.6	{13 204., 97 074.}	2994.23	1.33792	newly 5 weeks spec	4646.	1919.06	{70., 3967.}	68.0555	0.791151	newly 5 weeks cumul	8317.	53 991.7	{14 069., 116 087.}	551.251	0.679087	newly 64 weeks cumul	99 174.	57 097.6	{14 124., 120 439.}	51.6941	0.413679
11	newly 3 weeks spec	578.	2518.8	{0., 7530.}	430.716	1.5483	newly 3 weeks cumul	848.	33 446.5	{6776., 90 598.}	3845.62	1.43146	newly 5 weeks spec	4035.	1007.37	{0., 3915.}	85.4496	2.6567	newly 5 weeks cumul	6425.	35 964.8	{7026., 90 598.}	464.671	0.626415	newly 63 weeks cumul	99 600.	38 463.	{7052., 98 998.}	65.3832	0.596728
12	newly 3 weeks spec	936.	6560.49	{913., 14 863.}	605.36	0.689648	newly 3 weeks cumul	1372.	43 667.4	{12 157., 101 272.}	3082.76	1.35624	newly 5 weeks spec	5911.	2216.09	{52., 4351.}	74.3422	0.906712	newly 5 weeks cumul	9781.	49 587.5	{14 423., 119 611.}	411.957	0.605566	newly 64 weeks cumul	98 924.	53 896.7	{15 109., 124 853.}	54.5753	0.419315
13	newly 3 weeks spec	632.	5600.29	{262., 11 656.}	807.948	0.815292	newly 3 weeks cumul	864.	59 748.1	{13 764., 121 606.}	6815.29	1.70314	newly 5 weeks spec	4789.	1412.09	{1., 3169.}	81.5337	1.49099	newly 5 weeks cumul	7468.	63 910.3	{15 572., 121 698.}	762.916	0.865942	newly 65 weeks cumul	99 507.	65 732.5	{16 363., 121 698.}	44.991	0.346392
14	newly 3 weeks spec	1910.	1471.79	{252., 5979.}	101.047	0.64408	newly 3 weeks cumul	3449.	7846.87	{2262., 32 964.}	154.894	0.234462	newly 5 weeks spec	5421.	489.74	{17., 1592.}	90.9659	1.56134	newly 5 weeks cumul	12 064.	9185.84	{2691., 38 492.}	94.8927	0.528274	newly 58 weeks cumul	96 176.	10 010.	{2912., 39 593.}	89.592	1.22533
15	newly 3 weeks spec	1491.	1827.68	{113., 7021.}	164.048	0.822051	newly 3 weeks cumul	2425.	9285.9	{553., 17 242.}	324.605	0.35726	newly 5 weeks spec	6031.	527.36	{10., 1816.}	93.035	1.89853	newly 5 weeks cumul	11 560.	10 744.8	{702., 32 609.}	130.473	0.743441	newly 59 weeks cumul	97 278.	12 182.3	{814., 59 547.}	88.9947	1.46251
16	newly 3 weeks spec	1486.	965.64	{242., 1852.}	74.2638	0.506653	newly 3 weeks cumul	2628.	18 455.8	{7275., 44 032.}	607.816	0.714537	newly 5 weeks spec	4920.	172.82	{7., 197.}	96.4874	2.2139	newly 5 weeks cumul	10 361.	18 989.9	{7339., 44 135.}	107.902	0.272999	newly 62 weeks cumul	92 728.	19 350.2	{8151., 44 138.}	79.384	0.845821
17	newly 3 weeks spec	187.	6928.89	{11., 18 139.}	3634.67	1.43476	newly 3 weeks cumul	247.	46 500.3	{5942., 94 350.}	18 726.	2.07482	newly 5 weeks spec	2188.	3551.09	{0., 10 988.}	165.36	1.57926	newly 5 weeks cumul	3077.	54 832.2	{6989., 96 168.}	1683.56	1.09574	newly 68 weeks cumul	93 253.	66 074.9	{7314., 99 577.}	34.5321	0.306383
18	newly 3 weeks spec	162.	9424.06	{711., 20 181.}	5721.59	1.54768	newly 3 weeks cumul	222.	54 839.5	{11 760., 106 123.}	24 602.5	2.28182	newly 5 weeks spec	2807.	4629.07	{3., 10 318.}	136.989	1.07185	newly 5 weeks cumul	3654.	66 110.1	{14 082., 106 201.}	1709.25	1.17207	newly 67 weeks cumul	99 640.	76 795.8	{14 082., 108 631.}	27.4225	0.205662
19	newly 3 weeks spec	726.	1798.92	{277., 4202.}	186.231	0.382453	newly 3 weeks cumul	1060.	22 321.3	{8970., 60 501.}	2007.36	1.18997	newly 5 weeks spec	4054.	351.61	{7., 395.}	91.6909	1.83812	newly 5 weeks cumul	6766.	23 387.6	{9101., 61 403.}	248.141	0.424256	newly 62 weeks cumul	99 318.	23 867.	{9106., 61 442.}	75.9691	0.767516
20	newly 3 weeks spec	300.	10 829.3	{1016., 20 691.}	3513.42	1.37933	newly 3 weeks cumul	402.	48 142.6	{11 489., 96 241.}	11 876.4	1.94921	newly 5 weeks spec	3692.	5711.8	{22., 11 388.}	118.6	0.835653	newly 5 weeks cumul	5182.	61 943.6	{13 306., 96 926.}	1098.31	0.994586	newly 67 weeks cumul	98 887.	74 799.	{15 630., 105 313.}	27.4808	0.232906
21	newly 3 weeks spec	120.	8150.96	{75., 20 817.}	6710.97	1.57296	newly 3 weeks cumul	157.	40 022.1	{5263., 88 677.}	25 391.8	2.18858	newly 5 weeks spec	2417.	4123.88	{0., 11 823.}	164.579	1.36097	newly 5 weeks cumul	3205.	49 911.6	{6021., 94 013.}	1461.2	1.01433	newly 68 weeks cumul	99 034.	61 289.5	{6021., 96 684.}	39.0409	0.39922
22	newly 3 weeks spec	1137.	7102.8	{1002., 20 303.}	535.578	0.678367	newly 3 weeks cumul	1829.	52 056.8	{10 297., 117 464.}	2749.14	1.29262	newly 5 weeks spec	5958.	1901.58	{118., 3585.}	78.0111	0.927282	newly 5 weeks cumul	10 630.	57 461.3	{10 793., 120 658.}	449.18	0.645964	newly 64 weeks cumul	96 878.	60 553.7	{10 888., 120 854.}	51.4859	0.416096
23	newly 3 weeks spec	1235.	6894.37	{272., 12 536.}	484.338	0.762368	newly 3 weeks cumul	1958.	60 974.7	{20 418., 109 001.}	3014.13	1.4118	newly 5 weeks spec	6598.	2017.96	{0., 3983.}	77.8885	1.36009	newly 5 weeks cumul	11 490.	66 566.5	{23 743., 111 462.}	481.008	0.706201	newly 65 weeks cumul	88 617.	69 968.6	{31 677., 115 531.}	34.6978	0.218379
24	newly 3 weeks spec	102.	10 417.1	{847., 19 603.}	10 114.8	1.81617	newly 3 weeks cumul	138.	51 088.8	{11 853., 96 242.}	36 920.8	2.44933	newly 5 weeks spec	2027.	5519.38	{3., 12 181.}	223.839	0.905626	newly 5 weeks cumul	2665.	64 190.1	{13 424., 102 717.}	2308.63	1.29616	newly 67 weeks cumul	98 866.	78 732.4	{14 794., 105 879.}	24.4336	0.183518
25	newly 3 weeks spec	917.	2733.89	{0., 8563.}	284.123	1.31654	newly 3 weeks cumul	1410.	36 958.2	{6061., 88 480.}	2526.22	1.25132	newly 5 weeks spec	3941.	939.97	{0., 2890.}	87.6075	2.33613	newly 5 weeks cumul	7263.	39 395.	{7805., 92 873.}	451.71	0.645741	newly 63 weeks cumul	94 839.	41 811.4	{7851., 114 660.}	61.8826	0.574287
26	newly 3 weeks spec	2138.	262.84	{0., 480.}	91.1151	1.80635	newly 3 weeks cumul	3825.	5479.4	{418., 13 832.}	119.575	0.518411	newly 5 weeks spec	6408.	74.51	{0., 88.}	98.8372	3.05881	newly 5 weeks cumul	14 163.	5681.84	{460., 13 832.}	75.5867	0.787096	newly 61 weeks cumul	87 857.	5822.51	{478., 13 862.}	93.3727	1.52923
27	newly 3 weeks spec	1999.	617.24	{0., 481.}	108.315	1.78634	newly 3 weeks cumul	3536.	7554.95	{686., 15 485.}	162.053	0.419724	newly 5 weeks spec	6511.	133.12	{0., 82.}	98.5299	3.14524	newly 5 weeks cumul	13 890.	7978.54	{700., 15 485.}	78.875	0.626965	newly 61 weeks cumul	98 347.	8086.9	{704., 15 485.}	91.7772	1.40039
28	newly 3 weeks spec	310.	7683.66	{807., 20 692.}	2388.03	1.25184	newly 3 weeks cumul	474.	42 899.1	{7005., 95 746.}	8950.44	1.78008	newly 5 weeks spec	2971.	4374.22	{2., 11 226.}	123.581	1.03991	newly 5 weeks cumul	4384.	53 051.6	{9559., 97 311.}	1110.12	0.950814	newly 68 weeks cumul	89 828.	67 520.8	{12 644., 100 699.}	31.993	0.246792
29	newly 3 weeks spec	609.	6284.47	{300., 12 324.}	946.915	0.8721	newly 3 weeks cumul	951.	57 455.5	{19 764., 109 682.}	5941.89	1.67381	newly 5 weeks spec	3642.	1382.73	{2., 4116.}	74.5351	1.14281	newly 5 weeks cumul	6162.	61 733.3	{21 068., 120 330.}	904.521	0.919773	newly 65 weeks cumul	99 052.	63 192.8	{21 300., 122 563.}	46.4868	0.327797
30	newly 3 weeks spec	109.	7427.25	{130., 18 168.}	6727.86	1.59973	newly 3 weeks cumul	138.	41 971.1	{4549., 95 575.}	30 313.9	2.25227	newly 5 weeks spec	1649.	4401.42	{0., 11 830.}	245.687	1.24813	newly 5 weeks cumul	2255.	52 043.	{6551., 98 627.}	2209.99	1.18309	newly 68 weeks cumul	99 316.	66 132.6	{8551., 100 438.}	35.8333	0.341866
31	newly 3 weeks spec	3137.	1572.77	{272., 5230.}	87.021	0.728324	newly 3 weeks cumul	6051.	7884.69	{2599., 31 324.}	96.2299	0.304325	newly 5 weeks spec	6713.	568.6	{20., 1295.}	92.4731	1.55905	newly 5 weeks cumul	17 548.	9381.6	{3047., 39 247.}	81.0063	0.57525	newly 58 weeks cumul	87 424.	10 686.	{3297., 41 449.}	87.7768	1.12051
32	newly 3 weeks spec	1418.	6275.39	{907., 11 940.}	358.613	0.557969	newly 3 weeks cumul	2225.	51 761.7	{10 371., 118 381.}	2226.77	1.17389	newly 5 weeks spec	5244.	1348.95	{76., 3597.}	76.0597	0.933377	newly 5 weeks cumul	10 289.	55 828.7	{11 618., 121 522.}	452.171	0.62421	newly 64 weeks cumul	98 867.	57 982.4	{11 737., 121 775.}	52.9763	0.439923
33	newly 3 weeks spec	501.	5688.07	{66., 15 791.}	1063.69	1.05647	newly 3 weeks cumul	673.	37 716.4	{1640., 90 223.}	5514.73	1.49011	newly 5 weeks spec	5187.	3674.1	{0., 11 750.}	84.3004	1.39034	newly 5 weeks cumul	7427.	45 880.8	{1993., 94 129.}	540.311	0.785259	newly 68 weeks cumul	99 003.	60 637.8	{5174., 98 112.}	39.7001	0.465366
34	newly 3 weeks spec	1440.	7214.68	{920., 20 559.}	415.172	0.585185	newly 3 weeks cumul	2363.	51 842.5	{6494., 120 491.}	2095.38	1.17223	newly 5 weeks spec	6918.	1908.81	{51., 3636.}	77.2294	0.990246	newly 5 weeks cumul	12 627.	57 286.7	{8220., 123 641.}	364.515	0.572835	newly 64 weeks cumul	95 674.	60 530.6	{10 278., 123 906.}	50.4496	0.400869
35	newly 3 weeks spec	179.	7016.43	{42., 20 285.}	3840.49	1.31524	newly 3 weeks cumul	229.	39 300.3	{5792., 94 900.}	17 063.5	2.00489	newly 5 weeks spec	2514.	4224.08	{0., 13 087.}	166.569	1.46731	newly 5 weeks cumul	3425.	49 030.2	{7305., 94 966.}	1336.08	0.992539	newly 68 weeks cumul	95 744.	62 080.2	{7684., 100 316.}	38.2295	0.374687
36	newly 3 weeks spec	451.	6531.6	{969., 15 765.}	1349.23	0.969898	newly 3 weeks cumul	672.	44 317.2	{13 281., 103 924.}	6494.82	1.66479	newly 5 weeks spec	3163.	1693.18	{69., 3845.}	64.8479	0.684789	newly 5 weeks cumul	5077.	49 203.4	{14 546., 111 325.}	870.779	0.858318	newly 64 weeks cumul	99 501.	52 069.8	{14 840., 113 285.}	55.5098	0.440284
37	newly 3 weeks spec	142.	5643.8	{394., 11 445.}	3876.46	1.32438	newly 3 weeks cumul	189.	57 978.8	{10 319., 118 321.}	30 576.6	2.32417	newly 5 weeks spec	2178.	1499.55	{2., 3850.}	83.3448	1.09054	newly 5 weeks cumul	2952.	62 291.3	{11 674., 122 532.}	2016.27	1.21842	newly 65 weeks cumul	99 424.	64 477.1	{11 797., 122 532.}	45.3592	0.37438
38	newly 3 weeks spec	1570.	1162.65	{282., 1766.}	65.165	0.390852	newly 3 weeks cumul	2591.	20 670.6	{9001., 57 540.}	697.786	0.772374	newly 5 weeks spec	6266.	203.64	{7., 206.}	96.7501	2.11907	newly 5 weeks cumul	12 039.	21 322.8	{9109., 57 752.}	96.2518	0.219312	newly 62 weeks cumul	93 543.	21 586.7	{9116., 57 755.}	76.9233	0.766684
39	newly 3 weeks spec	955.	6337.39	{1047., 19 856.}	573.276	0.684537	newly 3 weeks cumul	1540.	41 366.6	{8276., 92 549.}	2586.49	1.24622	newly 5 weeks spec	4980.	1825.58	{102., 4070.}	71.3659	0.834251	newly 5 weeks cumul	8707.	46 471.2	{10 022., 105 167.}	442.341	0.626424	newly 64 weeks cumul	97 320.	49 546.3	{10 238., 111 633.}	55.9627	0.476854
40	newly 3 weeks spec	1152.	6525.87	{310., 13 235.}	493.766	0.682345	newly 3 weeks cumul	1821.	59 417.6	{21 564., 110 096.}	3163.95	1.40721	newly 5 weeks spec	6088.	1827.38	{2., 4127.}	81.9609	1.37607	newly 5 weeks cumul	10 814.	64 626.6	{24 130., 115 618.}	503.533	0.72397	newly 65 weeks cumul	99 384.	66 936.4	{25 032., 117 059.}	42.552	0.314151
41	newly 3 weeks spec	84.	6904.43	{750., 17 249.}	8127.96	1.6939	newly 3 weeks cumul	118.	37 707.5	{5521., 91 868.}	31 856.7	2.27161	newly 5 weeks spec	1333.	4257.91	{7., 11 595.}	293.357	1.01238	newly 5 weeks cumul	1781.	47 262.7	{5859., 92 007.}	2557.34	1.27215	newly 68 weeks cumul	96 330.	64 170.5	{7563., 98 707.}	36.4247	0.354721
42	newly 3 weeks spec	2385.	1663.84	{133., 973.}	126.797	0.903112	newly 3 weeks cumul	4744.	7894.76	{1484., 10 836.}	163.515	0.43965	newly 5 weeks spec	6151.	620.33	{11., 295.}	95.0039	1.82138	newly 5 weeks cumul	14 873.	9493.48	{1674., 10 852.}	111.065	0.771406	newly 59 weeks cumul	96 441.	10 656.8	{1691., 18 451.}	90.2255	1.42098
43	newly 3 weeks spec	942.	7149.43	{1005., 20 478.}	667.579	0.770854	newly 3 weeks cumul	1522.	47 267.5	{11 386., 107 124.}	3008.2	1.34716	newly 5 weeks spec	4281.	1873.88	{82., 3628.}	67.452	0.790837	newly 5 weeks cumul	7799.	52 685.7	{13 434., 124 232.}	579.188	0.71957	newly 64 weeks cumul	98 631.	55 601.6	{13 617., 124 445.}	55.4139	0.436259
44	newly 3 weeks spec	717.	7795.49	{946., 20 647.}	990.844	0.852549	newly 3 weeks cumul	1097.	49 638.5	{10 050., 115 530.}	4426.91	1.49653	newly 5 weeks spec	5449.	2044.28	{102., 3616.}	75.1334	0.864889	newly 5 weeks cumul	8791.	55 532.1	{10 233., 119 497.}	539.2	0.702771	newly 64 weeks cumul	99 299.	58 668.8	{10 626., 119 727.}	52.2683	0.454741
45	newly 3 weeks spec	514.	10 800.9	{2214., 21 126.}	2007.66	1.22572	newly 3 weeks cumul	734.	46 209.	{16 381., 76 933.}	6196.93	1.69646	newly 5 weeks spec	4552.	5171.19	{416., 10 639.}	70.8056	0.491624	newly 5 weeks cumul	6924.	58 864.7	{21 117., 97 483.}	753.808	0.880176	newly 66 weeks cumul	99 357.	72 757.8	{30 858., 111 795.}	33.9693	0.245504
46	newly 3 weeks spec	247.	7003.25	{398., 21 836.}	2750.69	1.28382	newly 3 weeks cumul	335.	45 885.4	{5592., 100 142.}	13 597.9	1.90117	newly 5 weeks spec	2717.	3879.06	{0., 12 405.}	145.856	1.34594	newly 5 weeks cumul	3855.	54 826.8	{6776., 100 219.}	1326.8	1.00466	newly 68 weeks cumul	99 095.	67 354.4	{7811., 100 607.}	34.6013	0.327099
47	newly 3 weeks spec	977.	3119.17	{0., 8629.}	308.734	1.48355	newly 3 weeks cumul	1597.	39 930.2	{6687., 98 053.}	2400.33	1.25077	newly 5 weeks spec	4864.	1028.16	{0., 3064.}	86.2327	2.44691	newly 5 weeks cumul	8755.	42 655.1	{6974., 107 747.}	393.247	0.58218	newly 63 weeks cumul	96 045.	44 800.3	{7001., 117 628.}	61.5608	0.496005
48	newly 3 weeks spec	364.	9243.59	{837., 21 211.}	2446.66	1.26649	newly 3 weeks cumul	527.	47 430.4	{9257., 95 449.}	8904.84	1.80512	newly 5 weeks spec	3183.	4182.75	{73., 9638.}	95.147	0.627084	newly 5 weeks cumul	4879.	57 786.1	{11 683., 105 780.}	1093.12	1.01278	newly 66 weeks cumul	97 052.	69 424.2	{21 400., 111 666.}	38.5304	0.30635
49	newly 3 weeks spec	1900.	7425.71	{1108., 15 875.}	308.267	0.52643	newly 3 weeks cumul	3162.	49 022.3	{11 191., 103 067.}	1451.84	1.05058	newly 5 weeks spec	7611.	2078.5	{149., 4666.}	76.3913	0.952555	newly 5 weeks cumul	14 764.	54 899.8	{13 715., 118 119.}	281.326	0.498688	newly 64 weeks cumul	95 259.	58 050.2	{14 067., 118 455.}	51.2457	0.39537
50	newly 3 weeks spec	943.	5940.61	{307., 11 508.}	556.012	0.777127	newly 3 weeks cumul	1533.	56 370.9	{17 766., 110 472.}	3580.22	1.44348	newly 5 weeks spec	5024.	1336.95	{2., 3259.}	76.2199	1.29594	newly 5 weeks cumul	8966.	60 452.9	{18 829., 114 762.}	584.348	0.792018	newly 65 weeks cumul	98 082.	62 778.8	{19 088., 116 402.}	45.4447	0.360458
